# Presence of *Leptospira* spp. in a Mosaic of Wetlands Used for Livestock Raising under Differing Hydroclimatic Conditions

**DOI:** 10.1128/aem.01971-22

**Published:** 2023-05-22

**Authors:** Yosena T. Chiani, Paulina Jacob, Gisela Mayora, Diego S. Aquino, Rubén D. Quintana, Leticia Mesa

**Affiliations:** a Laboratorio Nacional de Referencia de Leptospirosis, Instituto Nacional de Enfermedades Respiratorias Dr. E. Coni, Administración Nacional de Laboratorios e Institutos de Salud (ANLIS Dr. C.G. Malbran), Santa Fe, Argentina; b Laboratorio de Leptospirosis, Facultad de Bioquímica y Ciencias Biológicas, Universidad Nacional del Litoral, Santa Fe, Argentina; c Instituto Nacional de Limnología (INALI-CONICET-UNL), Ciudad Universitaria, Santa Fe, Argentina; d IIIA-UNSAM-CONICET, Instituto de Investigación e Ingeniería Ambiental, Escuela de Hábitat y Sostenibilidad, Buenos Aires, Argentina; e Consejo Nacional de Investigación en Ciencia y Tecnología, Buenos Aires, Argentina; Unversidad de los Andes

**Keywords:** *Leptospira* spp., physical, chemical, and hydrometeorological conditions, wetlands

## Abstract

Knowledge about the life cycle and survival mechanisms of leptospires in the environment is scarce, particularly regarding the environmental factors associated with their presence in ecosystems subject to livestock farming, where precipitation, seasonal floods, and river overflows could act as facilitators of leptospire dispersion. This study aimed to identify and study the presence of *Leptospira* spp. in the Lower Delta of the Paraná River and describe the physical, chemical, and hydrometeorological conditions associated with their presence in wetland ecosystems impaired by livestock raising intensification. Here, we show that the presence of *Leptospira* was determined mainly by water availability. We detected the species Leptospira kmetyi, L. mayottensis, and L. fainei and successfully cultured the saprophytic species L. meyeri from bottom sediment, suggesting the association of leptospires with microbial communities of the sediment’s biofilm to enhance its survival and persistence in aquatic environments and adapt to changing environmental conditions. Knowledge of *Leptospira* sp. diversity in wetlands and the impact of climate variability on the transmission of these organisms is crucial for predicting and preventing leptospirosis outbreaks in the context of human health.

**IMPORTANCE** Wetlands are environments that are often conducive to the survival and transmission of *Leptospira* because they provide a suitable habitat for the bacteria and are often home to many animal species that can act as reservoirs for leptospirosis. Bringing humans and animals into closer contact with contaminated water and soil and increased frequency and intensity of extreme weather events may further exacerbate the risk of leptospirosis outbreaks, which is mostly relevant in the context of climate change and a widespread intensification of productive activities, particularly in the Lower Delta of the Paraná River. The detection of leptospiral species in wetland ecosystems impaired by livestock raising intensification can help to identify propitious environmental factors and potential sources of infection, develop preventive measures, and plan for appropriate responses to outbreaks, ultimately improving public health outcomes.

## INTRODUCTION

Leptospirosis is one of the most important zoonoses worldwide, affecting both developing and developed countries (1, 2). Leptospirosis arises from the infection of spirochaetes of the genus *Leptospira*. The epidemiology of leptospirosis and the ecology of *Leptospira* spp. are particularly complex given the genetic diversity of the genus, which involves 82 species segregated into two phylogenetic clades. The saprophytic clade includes species isolated in the natural environment and not responsible for infections, while the pathogenic clade includes all the species responsible for infections in humans and/or animals, plus environmental species for which the virulence status has not been proven (3, 4).

Mainly through their skin and mucous membranes, humans and animals become infected when they frequent a *Leptospira*-contaminated environment (5). Even though infected cattle may not show any clinical sign of disease, bacteria might be excreted through their urine, and therefore, cattle may play a critical role in spreading the infection to other susceptible animals and human populations (5–8).

Knowledge about the life cycle and survival mechanisms of leptospires in the environment remains scarce (6), particularly regarding the environmental factors associated with their presence in wetland ecosystems subject to livestock farming (9). Genomic studies suggest that some pathogenic *Leptospira* species evolved from an environmental *Leptospira* found in water or mud. However, there seems to be some evolutionary variation among pathogenic leptospires; some species (such as Leptospira interrogans) have retained genes associated with environmental survival, whereas other parasitic leptospires (such as Leptospira borgpetersenii) seem to have lost these genes (10, 11). The ability of *Leptospira* organisms to remain viable and infectious across different environments and substrates (such as soil, sediment, and water) suggests that these bacteria have fast-acting, sensitive regulatory systems that allow them to adapt to various environmental challenges (9).

The distribution and prevalence of *Leptospira* species can vary depending on several factors, including geographical location, climate, host species, and human behavior. These factors can contribute to differences in the genetic diversity and distribution of *Leptospira* species between neighboring countries or even within the same country. For instance, studies suggest that there are even significant differences in the distribution and prevalence of *Leptospira* species between the neighboring countries Argentina and Uruguay (12), suggesting that different *Leptospira* species may be more prevalent in certain regions than others.

Higher seasonal rainfall and warmer temperature might increase *Leptospira* viability (13, 14). Precipitation events would increase runoff to water bodies, constituting a relevant factor in the dissemination and incorporation of bacterial species (6, 15, 16). Thus, precipitation, seasonal floods, and river overflows could act as facilitators of leptospire dispersion in wetland ecosystems. In this context, extraordinary hydrometeorological events would increase river streamflow and dilute the concentration of pathogenic *Leptospira* spp., subsequently flushing all the microbes, including pathogenic *Leptospira* spp., from riparian areas into surface waters (17). Furthermore, in wetland ecosystems, changes in hydrometeorological conditions are usually associated with modifications in water quality variables such as pH, dissolved oxygen concentration, and nutrient concentrations (18, 19). Several studies postulated the persistence of *Leptospira* spp. in different types of aquatic systems with slightly alkaline pH, high oxygen, low concentrations of heterotrophic bacteria, and low salt concentrations (2, 6, 15, 20, 21). However, no studies on the environmental factors and determinants that affect the presence of *Leptospira* in wetland systems have been reported. This scarcity of knowledge contributes to our insufficient understanding of basic aspects of leptospirosis epidemiology.

The difficulty of isolating pathogenic *Leptospira* species from environmental samples constitutes a major impediment to assessing the environmental risk of leptospirosis, mostly because nonpathogenic leptospires outgrow pathogenic strains in culture (22). The detection of pathogenic *Leptospira* in water samples is also difficult due to dilution of the pathogen as well as to the potentially higher number of other bacterial species, which usually contaminate culture media. To date, there is no standard protocol for culturing the pathogenic species of *Leptospira* from environmental soil or water samples. Furthermore, only a few reports have described the isolation of pathogenic *Leptospira* spp. from environmental samples (23–25). However, the increasing use of molecular methods overcomes some limitations inherent in culture- and animal-based methods and provides quantitative information about the concentration of leptospires in contaminated waters (15, 26).

The Paraná River Delta is a highly biodiverse mosaic of wetland ecosystems in South America. It provides numerous ecosystem functions and services, such as reduction in water flow and turbulence, increased short- and long-term water retention capacity and regulation of evapotranspiration, provision of forage for livestock and habitat for wildlife species, and climate change mitigation (27, 28). Particularly in the noninsular portion of the Lower Delta, extensive livestock raising has been, traditionally, the most widespread anthropic activity. Nevertheless, the expansion of soybean production in the late 20th century has forced the relocation of cattle toward marginal sites for agriculture, such as the Paraná Delta region (29). In consequence, cattle numbers have significantly increased accompanied by an unrestricted and widespread implementation of water management infrastructure (30). In these wetlands, precipitation and a high water level would facilitate the introduction of *Leptospira* species eliminated from urine of infected cattle to water bodies. In the context of intensification of livestock-raising activity, this study aimed to identify and study the presence of *Leptospira* spp. in the Lower Delta of the Paraná River (LDPR) across three periods differing in their hydroclimatic conditions and water quality variables. We hypothesize that the presence of *Leptospira* in wetland ecosystems is higher during periods of higher water availability.

## RESULTS

### Hydrometeorological variables.

**FIG 1:**
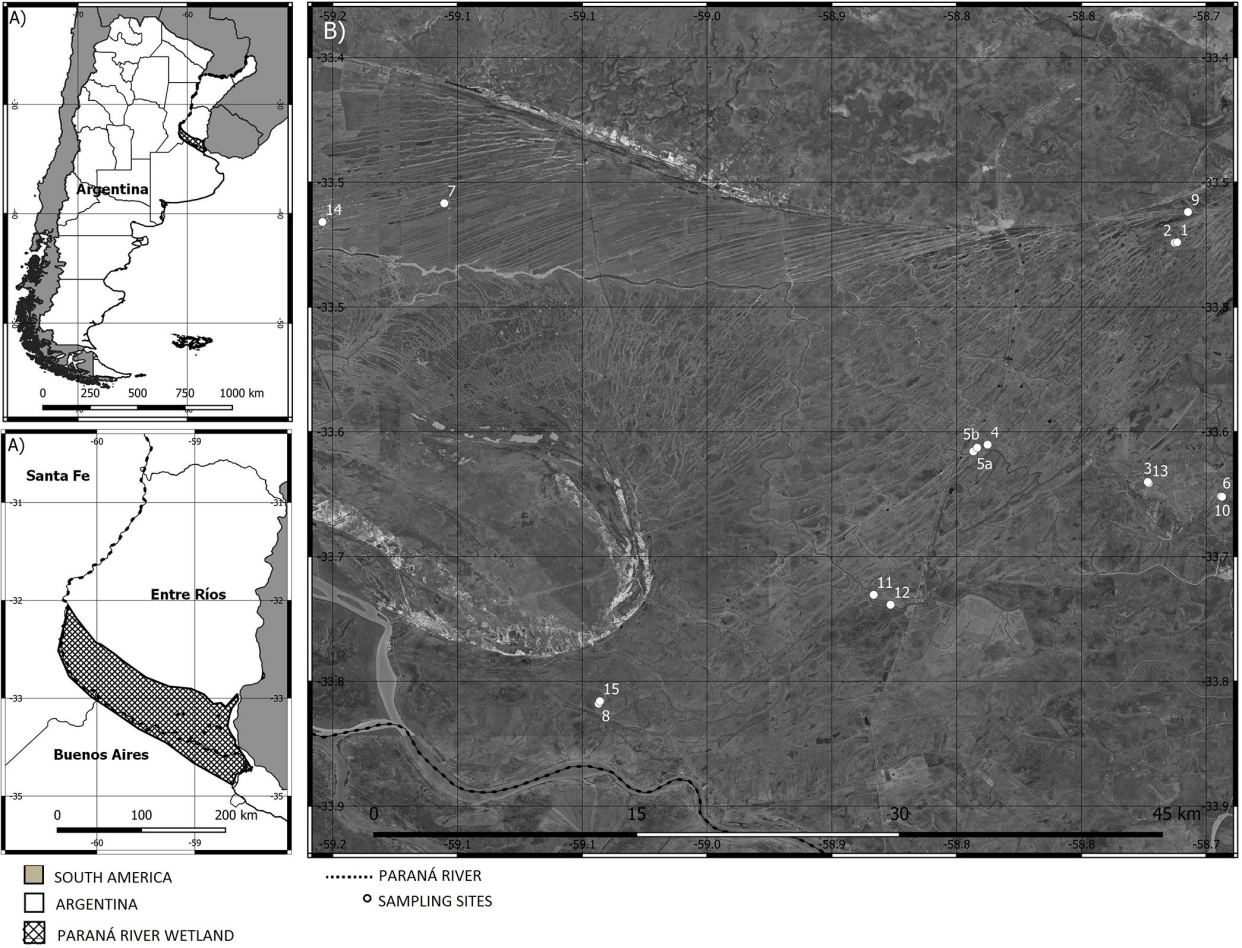
(A) Location of the study area in South America and Argentina (top) and amplified image of the Paraná River wetland in Entre Ríos Province (bottom). (B) Location of the sampled sites. Source: Instituto Geográfico Nacional.

**FIG 2:**
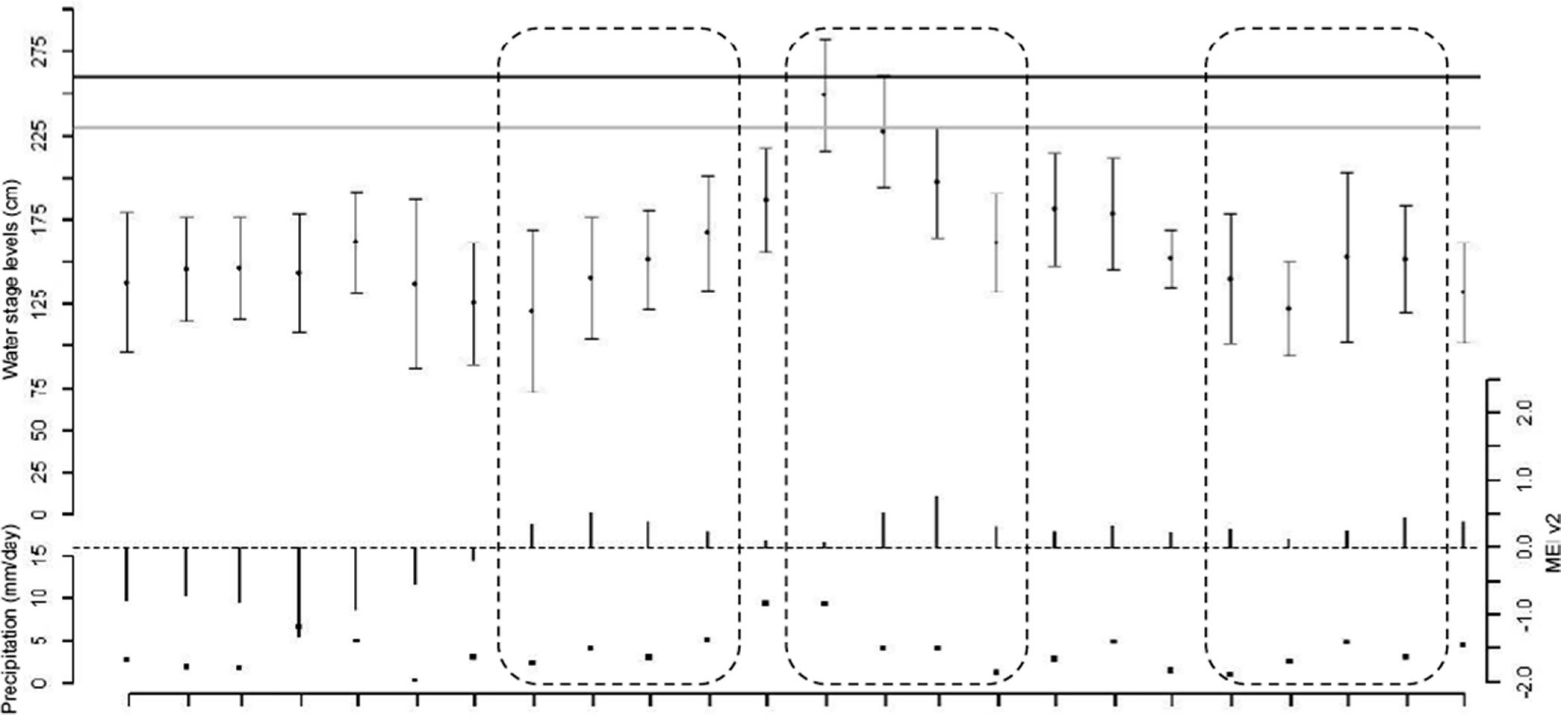
Time series of hydroclimatic variables as indicators of water availability in the study area. Monthly time series values for Paranacito River water stage and its standard deviations (top; black dots and error bars) as well as its evacuation and alert levels (black and gray horizontal lines, respectively), MEI.v2 (middle; vertical black bars), and rainfall (bottom, black squares).

A map of the study area is provided in Fig. 1. In general, no significant El Niño or La Niña events were identified between January 2018 and December 2019. Land surface temperatures exhibited a strong seasonal pattern with no particular anomalies. Nevertheless, multivariate ENSO index (MEI) v2 values were lower and below zero during most of 2018 and higher and above zero during 2019. These values were consistent with lower water stage levels of the Paranacito River in 2018 and higher levels in 2019, including a series of extraordinary overflow events that occurred between January and March 2019 (Fig. 2). With regard to precipitation, the observed values exhibited a mild seasonal pattern. However, several extraordinary precipitation events occurred in December 2018 and January 2019 (Fig. 2).

Thus, three different hydroclimatic scenarios were identified: a drier scenario in November 2018, consisting of mostly lower precipitation values and lower yet increasing water stage levels (August 2018 to November 2018); a more humid period during April 2019, characterized by higher precipitation rates and higher yet decreasing water stage levels (January 2019 to April 2019); and a much drier period in November 2019, characterized by lower and sustained Paranacito River water stage levels and lower precipitation rates, which translated into widespread lack of water availability (August 2019 to November 2019).

### *Leptospira* isolation, detection, and identification.

Thirty-four water samples and 12 sediment samples from 15 sampling sites were collected in marshlands and ditches during the three sampling periods. With regard to water samples, the pathogenic species Leptospira kmetyi (S1 and S5a) and L. mayottensis (S2), as well as the intermediate species L. fainei (S5b), were identified in four freshwater marshlands during April 2019 (humid period). In addition, *L. kmetyi* was detected in a ditch (S12) during November 2018 (dry period) (Table 1). Regarding culture from water samples, 44.1% (15 samples from a total of 34) showed no development of leptospires, whereas the remaining water samples (55.9%) were contaminated with a higher number of bacteria. In sediment samples, the saprophytic species L. meyeri was successfully isolated from a single sample relative to a freshwater marshland (S1) in November 2019 (much drier period). The remaining sediment cultures were contaminated.

**TABLE 1:** Culture and PCR results of water and sediment samples from marshes and ditches^*a*^

Sample	Site	Water samples									Sediment samples		
		November 2018			April 2019			November 2019			November 2019		
		Culture	PCR	Species (% similarity)	Culture	PCR	Species (% similarity)	Culture	PCR	Species (% similarity)	Culture	PCR	Species (% similarity)
1	Marsh	No development	ND		Contaminated	Detected	*L. kmetyi* (81.13)	No development	ND		Development	ND	*L. meyeri* (99.68)
2	Marsh	No development	ND		No development	Detected	*L. mayottensis* (91.55)	No development	ND		Contaminated	ND	
3	Marsh	No development	ND		Contaminated	ND		No sample	No sample		No sample	No sample	
4	Marsh	No development	ND		No sample	No sample		Contaminated	ND		Contaminated	ND	
5a	Marsh	No development	ND		Contaminated	Detected	*L. kmetyi* (88.1)	No development	ND		Contaminated	ND	
5b	Marsh	No development	ND		Contaminated	Detected	*L. fainei* (78.24)	No sample	No sample		No sample	No sample	
6	Marsh	No development	ND		Contaminated	ND		No development	ND		Contaminated	ND	
7	Marsh	No sample	No sample		No sample	No sample		Contaminated	ND		Contaminated	ND	
8	Marsh	No sample	No sample		Contaminated	ND		No sample	No sample		No sample	No sample	
9	Marsh	No sample	No sample		No sample	No sample		Contaminated	ND		Contaminated	ND	
10	Ditch	No development	ND		No sample	No sample		No sample	No sample		No sample	No sample	
11	Ditch	Contaminated	ND		Contaminated	ND		Contaminated	ND		Contaminated	ND	
12	Ditch	Contaminated	Detected	*L. kmetyi* (84.4)	Contaminated	ND		Contaminated	ND		Contaminated	ND	
13	Ditch	No development	ND		Contaminated	ND		No development	ND		Contaminated	ND	
14	Ditch	No sample	No sample		No sample	No sample		Contaminated	ND		Contaminated	ND	
15	Ditch	No sample	No sample		Contaminated	ND		Contaminated	ND		Contaminated	ND	

a Water samples from marshes and ditches were collected during November 2018 (dry period), April 2019 (humid period), and November 2019 (much drier period), and sediment samples were obtained in November 2019. “Contaminated” indicates cultures contained a higher number of bacteria other than *Leptospira.* “No sample” indicates that no samples were collected for *Leptospira* analysis due to the impossibility of accessing the site because of flooding or desiccation. “ND” indicates that 16s rRNA gene of *Leptospira* was not detected by PCR.

### Comparison of water quality variables among sampled periods.

**FIG 3:**
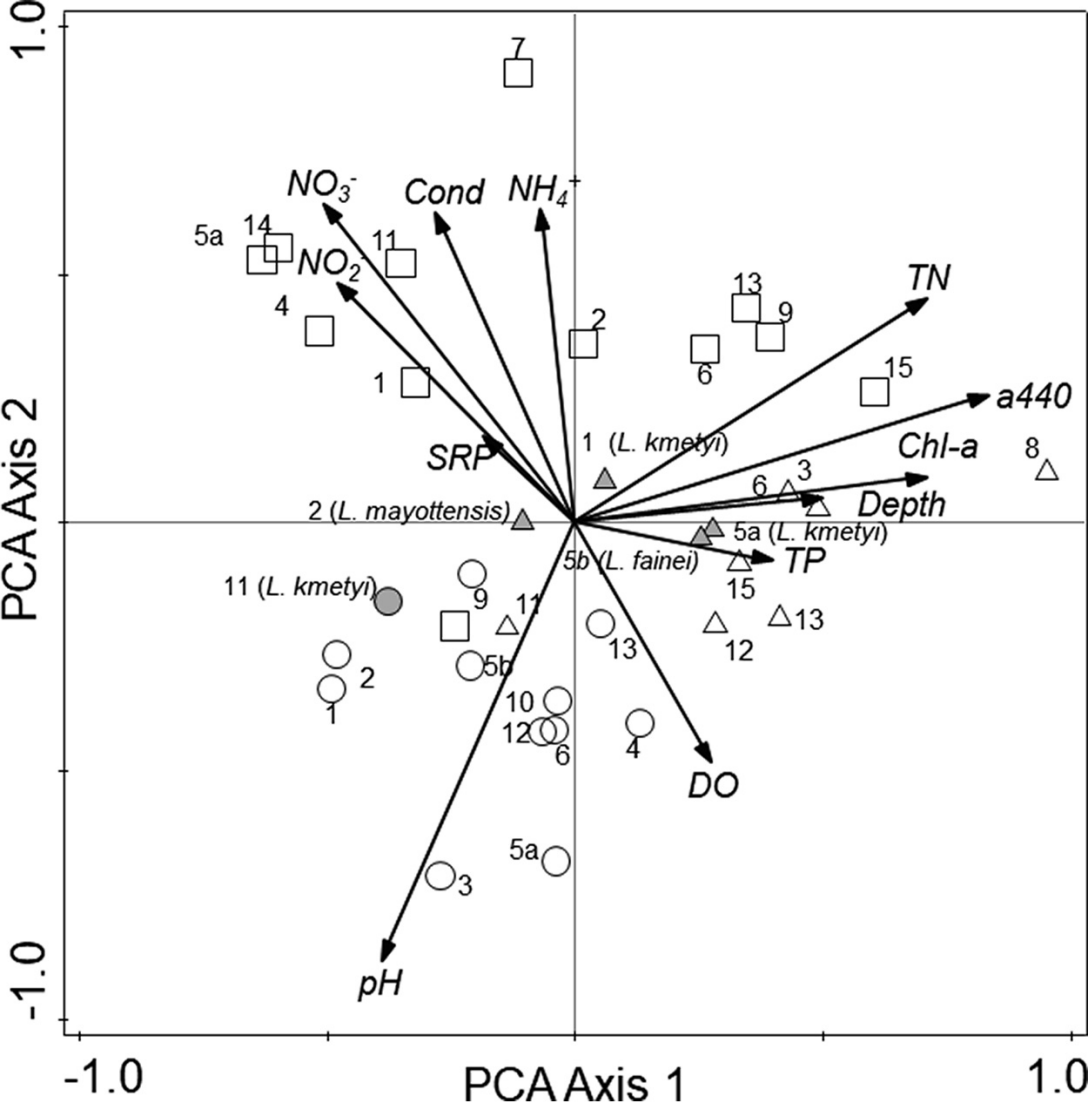
Principal-component analysis biplot of axes 1 and 2 showing the main variation patterns of water quality among the marshlands and ditches sampled during November 2018 (circles), April 2019 (triangles), and November 2019 (squares). Gray symbols indicate the presence of a *Leptospira* sp., and the species name is given in parentheses. Information on sampling sites is available in Table 1.

Most physical and chemical variables exhibited a strong spatial and temporal variability (Table 2; Fig. 3). The first two principal components (PC1 and PC2) of the principal-component analysis (PCA) explained 47.4% of the total variability. The score plot of PC1 versus PC2 showed that water samples were grouped in terms of sampling periods. November 2018 (dry period) (Fig. 3, lower left quadrant) was mostly associated with higher pH values, whereas April 2019 (humid period) (Fig. 3, upper and lower right quadrants) was mainly associated with higher depth and concentrations of chromophoric dissolved organic matter (CDOM), total phosphorus (TP), total nitrogen (TN), and chlorophyll *a*. Last, November 2019 (much drier period) (Fig. 3, upper left and right quadrants) was strongly related to higher concentrations of dissolved inorganic nutrients and conductivity but lower dissolved oxygen concentration (Fig. 3).

**TABLE 2:** Values of chemical and physical variables in each aquatic environment of the Paraná Delta^*a*^

Period	Site	*A*_440_ (m^−1^)	TN (μg L^−1^)	TP (μg L^−1^)	NH_4_^+^ (μg L^−1^)	SRP (μg L^−1^)	NO_3_^−^ (μg L^−1^)	NO_2_^−^ (μg L^−1^)	pH	DO (mg L^−1^)	Cond (μS cm^−1^)	Temp (°C)	Depth (cm)	Chl *a* (μg L^−1^)
Nov18	1	4.6	1183	226	138	74	<QL	1.5	7.7	7.96	228	21.3	61	4.0
	2	6.0	1318	91	96	21	<QL	<QL	7.7	7.76	1361	21.4	70	3.0
	3	21.2	1712	747	<QL	156	<QL	<QL	7.9	4.65	93	20.9	30	17.7
	4	13.6	3087	54	<QL	0	<QL	<QL	6.5	2.05	173	22.0	67	2.4
	5a	8.2	1290	386	<QL	92	<QL	<QL	8.0	0.96	101	22.2	50	10.7
	5b	11.8	1738	358	38	86	<QL	<QL	6.9	2.81	112	25.1	12	4.0
	6	10.3	2578	829	25	140	<QL	<QL	7.4	9.62	126	24.1	50	8.0
	9	19.9	2165	500	295	55	<QL	4.5	7.7	4.88	167	25.1	53	8.9
	10	10.5	2895	441	12	75	<QL	<QL	7.4	2.56	162	21.0	100	5.8
	11	7.9	1687	917	180	243	<QL	<QL	8.0	<DL	508	19.9	100	4.6
	**12**	**8.7**	**1222**	**391**	**42**	**29**	**<QL**	**<QL**	**7.8**	**2.92**	**184**	**22.5**	**80**	**60.1**
	13	12.4	2081	1483	66	239	<QL	<QL	7.2	2.72	151	22.5	48	27.0

Apr19	**1**	**10.5**	**1475**	**523**	**27**	**258**	**<QL**	**<QL**	**6.6**	**0.94**	**846**	**21.3**	**87**	**40.3**
	**2**	**11.1**	**1763**	**81**	**34**	**21**	**<QL**	**<QL**	**6.7**	**0.61**	**576**	**23.7**	**60**	**9.5**
	3	23.6	5800	170	27	24	<QL	<QL	6.0	6.95	183	28.3	35	14.2
	**5a**	**10.8**	**7388**	**435**	**89**	**27**	**<QL**	**<QL**	**6.5**	**10.80**	**247**	**25.2**	**72**	**14.6**
	**5b**	**10.5**	**3484**	**649**	**60**	**36**	**<QL**	**<QL**	**6.5**	**6.06**	**294**	**27.7**	**55**	**35.2**
	6	17.3	3781	345	28	19	<QL	<QL	5.9	4.43	133	21.2	62	14.4
	8	47.1	3563	455	27	27	<QL	<QL	5.7	6.10	72	23.5	60	176.9
	11	7.4	1806	363	24	202	<QL	<QL	6.8	9.33	391	21.1	40	7.8
	12	8.9	1703	658	25	17	<QL	<QL	6.3	11.80	212	21.4	80	15.7
	13	17.5	6775	854	22	229	<QL	<QL	6.5	6.52	37	26.8	47	20.7
	15	29.7	4750	516	35	51	<QL	<QL	6.4	9.44	142	24.8	32	6.1

Nov19	**1**	**14.0**	**1909**	**1236**	**112**	**1235**	**<QL**	**1.0**	**6.9**	**2.02**	**3870**	**24.9**	**40**	**4.4**
	2	18.8	6041	1277	83	1182	<QL	<QL	6.7	<DL	1748	23.5	70	10.4
	4	11.1	2169	130	77	128	119	1.0	6.6	<DL	366	26.3	35	2.1
	5a	7.2	1655	170	89	65	232	0.8	6.3	<DL	953	24.1	35	1.0
	6	12.0	1519	63	65	18	<QL	<QL	5.2	1.44	257	20.6	50	6.3
	7	17.0	22861	226	393	27	182	5.5	6.3	<DL	797	26.6	50	25.5
	9	44.2	19348	575	641	147	<QL	<QL	6.75	4.00	649	29.8	40	75.5
	11	8.5	2738	638	28	288	159	1.7	6.3	<DL	740	20.4	58	5.8
	12	8.1	3195	125	67	109	<QL	<QL	6.9	7.47	185	31.3	19	4.7
	13	17.4	5946	166	62	152	<QL	<QL	5.3	<DL	97	20.7	35	4.9
	14	13.8	7728	930	141	78	2396	74.9	7.9	9.03	1570	29.4	55	51.1
	15	33.8	6275	188	15	23	<QL	<QL	6.2	<DL	360	22.5	160	57.6

a The Paraná Delta was sampled in November 2018 (Nov18), April 2019 (Apr19), and November 2019 (Nov19). *A*_440_, absorption coefficient of CDOM at 440 nm; TN, total nitrogen; TP, total phosphorus; SRP, soluble reactive phosphorus; DO, dissolved oxygen; Cond, conductivity; Chl *a*, chlorophyll *a*; QL, quantitation limit; DL, detection limit. Rows corresponding to samplings where *Leptospira* was detected in water are in bold.

Repeated-measures analysis of variance (ANOVA) showed that November 2018 significantly differed from April 2019 (*F* = 58.87, *P* < 0.001) and November 2019 (*F* = 27.75, *P* < 0.01) due to higher pH values. On the other hand, a significant increase in TN (*F* = 21.08, *P* < 0.01) and CDOM evaluated as the absorption coefficient (m^−1^) at 440 nm (*A*_440_) (*F* = 6.30, *P* < 0.05) was observed in April 2019 in comparison to November 2018. In contrast, November 2019 showed significantly higher values of conductivity and lower dissolved oxygen than April 2019 (*F* = 25.38, *P* < 0.001, and *F* = 10.82, *P* < 0.05, respectively) and November 2018 (*F* = 7.14 and 8.35, *P* < 0.05).

## DISCUSSION

This is the first study detecting and identifying *Leptospira* species across a mosaic of heavily altered wetlands of the Paraná River system, in the context of intensified livestock raising. We detected the pathogenic species *L. kmetyi* and *L. mayottensis*, the intermediate species *L. fainei*, and the saprophytic species *L. meyeri*.

Our results showed that the presence of *Leptospira* species was strongly associated with the varying hydrometeorological conditions and, thus, mostly determined by water availability. Our findings were complemented by a description and assessment of the physical and chemical properties of water samples in freshwater marshlands and anthropogenic ditches, in the context of different hydrometeorological periods. Leptospira meyeri and *L. kmetyi* had already been found in other aquatic systems in subtropical and tropical regions around the world (13, 24, 31–33). Our results identify *L. mayottensis* for the first time in a wetland subjected to cattle use in Argentina. This genomospecies has been isolated from blood of leptospirosis patients in Mayotte (34) and from environmental samples in New Zealand (35).

Our results support the idea that climatic factors such as heavy rainfall and flooding can increase the risk of leptospirosis outbreaks by promoting the growth and spread of *Leptospira*, particularly during El Niño southern oscillation (ENSO) events (14, 36). In accordance with our hypothesis, a greater number of *Leptospira* species, and particularly pathogenic and intermediate species, were detected in water samples from the humid period (April 2019) than in samples from the drier periods (November 2018 and 2019). The aforementioned findings imply a positive relationship between *Leptospira* incidence and water availability. In the study area, cattle graze and urinate across the full extent of the topographical gradient, including seasonally flooded freshwater marshlands that usually dominate in the lower topographical settings. These areas constitute an important source of water for cattle as long as the hydrologic regimen and hydrological connectivity are preserved. In this context, river overflows and rainfall events play an important role in water replenishment of topographically lower areas (37). Periods of higher precipitation rates and increased water stage levels would flush *Leptospira* from topographically higher areas to water bodies, resulting in a higher incidence of these bacteria. Several studies carried out in tropical and subtropical regions have revealed the association between the occurrence of *Leptospira* in water bodies and periods of heavy rainfall and flooding as well (15, 36, 38–41).

Differing hydroclimatic scenarios in the study area had previously been associated with changes in physicochemical conditions of water (19). Furthermore, the results of this study showed that the highest values of CDOM evaluated as *A*_440_, TP, TN, chlorophyll *a*, and depth occurred during the humid period (April 2019). This period exhibited a higher presence of *Leptospira* in water, despite the fact that pH and dissolved oxygen conditions were unfavorable for its survival. This suggests that organic matter from nonpoint sources, including animal waste, was dragged by rains and increased water stage levels into freshwater marshlands and ditches, along with the microorganisms that it might contain, such as *Leptospira*. This result agrees with other studies of wetlands of the Paraná River system that associate wet conditions with the mobilization of organic matter from higher topographical areas into aquatic systems (42). In wetland ecosystems, bacterial mineralization of organic matter consumes oxygen and releases carbon dioxide (43), which might explain the generally low values of dissolved oxygen and slightly acidic waters in the study sites. In this regard, our results suggest that hydroclimatic conditions favoring *Leptospira* transport from terrestrial to aquatic habitats were more important drivers of their presence than abiotic variables able to affect bacterial survival (e.g., pH and dissolved oxygen).

During November 2019 (the driest evaluated period), *L. meyeri* was isolated from a sediment sample of a freshwater marshland subjected to livestock raising. This result suggests that *L. meyeri* could survive and persist in unfavorable conditions through its association with microbial communities of the sediment’s biofilm, which would increase its ability to adapt to changing environmental conditions (9, 15, 23, 44, 45). Biofilms are believed to share a protector effect (20, 46), contributing to the persistence and long-term survival of leptospires in adverse aquatic and wetland environments (15, 44). It has been observed that leptospiral biofilm has a 5- to 6-fold increase in antibiotic resistance in all the strains used. It is tempting to speculate that biofilms may protect *Leptospira* against other toxic compounds in the environment (11).

With regard to the difficulty in properly culturing pathogenic species of *Leptospira* and their low growth rate, previous research found that it takes at least 10^6^ UFC/mL to obtain an isolate (22). Saprophytic *Leptospira* spp. are most frequently isolated from environmental samples because they are common inhabitants of the environment and grow faster (47–49), whereas pathogenic and intermediate *Leptospira* spp. are able to survive but not multiply in the environment (6, 22). In agreement with this, we successfully isolated the nonpathogenic specie *L. meyeri*, but most sediment and water samples were contaminated with others microorganisms despite the use of the combination of 5-fluorouracil with sample prefiltration through 0.22-μm-pore-size filters. These results suggest that future strategies might include more antimicrobial agents in the culture medium (6, 50).

It is well known that the presence of pathogenic and intermediate *Leptospira* spp. constitutes a risk to human health and wildlife. In cattle, leptospirosis has been identified as one of the major causes of reproductive failure, including infertility. Increases in the number of services per conception, longer calving intervals, abortion, the occurrence of stillbirths and weak offspring, leading to significant economic hazards, have been observed as well (51). In this context, veterinarians, agriculture workers, abattoir workers, farm workers, fishers, and hunters can become infected during occupational activities that involve contaminated water. In addition, there is also a significant risk of exposure associated with recreational activities, including swimming, kayaking, canoeing, and triathlons, as well as military training exercises (52). Considering that human settlements and livestock numbers will probably increase over the years (30), measures should be considered to prevent the transmission of leptospirosis from its habitat to livestock or humans, and vice versa. Particularly in wetlands of the Paraná River Delta, the aforementioned considerations should be made while taking into account the periodicity of the flood pulse, as well as anomalous seasonal patterns of precipitation during extraordinary meteorological events such as El Niño southern oscillation.

The results presented in this study reinforce the positive association between humid conditions and pathogenic *Leptospira* in wetland ecosystems in a context of anthropogenic land use intensification. Informing decision makers of the seasonal hydrometeorological drivers of *Leptospira* presence and abundance should be the first step in preventing, forecasting, and planning control of leptospirosis, which affects not only humans in the contexts of recreational and productive activities but also animal health.

### Conclusion.

We describe the physical, chemical, and hydrometeorological conditions that could be associated with the presence of *Leptospira* in wetland ecosystems impaired by intensified livestock raising. The results of our study reinforce the idea that leptospires thrive and survive in both natural and heavily modified wetlands, such as freshwater marshlands and anthropogenic ditches, respectively. In turn, evidence supports the idea that *Leptospira* adapts to unfavorable and diverse environmental conditions.

Detection of pathogenic and intermediate *Leptospira* in these wetlands during relatively humid periods could imply an increased human health risk, especially for those who come into contact with contaminated water and sediment in seasonally flooded areas. To help expand the current knowledge regarding leptospirosis survival as well as the spatial and temporal distribution of leptospires in the environment and animals, further studies should aim to describe the physicochemical characteristics not only of surface waters but also of bottom sediments of wetlands (including artificial water bodies) and consider the associations between *Leptospira* and biofilms.

Knowledge of *Leptospira* diversity in wetlands and the impact of climate variability on their transmission is crucial for predicting and preventing leptospirosis outbreaks in the context of human health. It can help identify potential sources of infection, develop preventive measures, and plan for appropriate responses to outbreaks, ultimately improving public health outcomes.

## MATERIALS AND METHODS

### Study area.

The study area is located in the noninsular portion of the Lower Delta of the Paraná River, which is part of the Delta Region (17,500 km^2^), in Argentina (South America) (Fig. 1). The Paraná Delta is a vast and highly biodiverse macromosaic of wetlands. It is characterized by a high environmental heterogeneity, mainly due to its geomorphological and hydrological complexity (27, 28, 53). The noninsular portion of the LDPR (−33° 45′S; 58° 51′W) exhibits conspicuous geomorphological patterns, which are a result of marine ingressions and regressions that occurred in the Holocene and are still affected by ongoing fluvial processes (29, 53). These patterns have been characterized as several landscape units differing in their hydrological regimens as well as in their geomorphological settings and land cover patterns (28, 54). Its hydrologic regimen is affected not only by local rains but also by seasonal and extraordinary river overflows from the Paranacito, Paraná, Gualeguay, and Uruguay rivers, whose effects are spatially variable and dependent on hydrogeomorphological and anthropogenic features (37, 54).

The climate is subhumid; the average (2001–2020) mean annual temperature and accumulated annual precipitation were 21.9°C and 1,358 mm, respectively (37). Normally, the study area exhibits a mild seasonal pattern with regard to temperature and precipitation. In addition, the area presents an important interannual variability due to ENSO, as well as high seasonal variability in precipitation and river water stage levels based on local hydroclimatic factors (37). As livestock raising has expanded and intensified, most of these patterns have been altered due to the unrestricted development of water management infrastructure, such as polders, embankments, and channelizations (30, 55). The study area is entirely affected by livestock raising. Different cattle management practices occur across the full extent of the topographical gradient, which implies direct contact with mostly permanently flooded freshwater marshlands that comprise the most important wetland vegetation physiognomies. In addition, recreational activities such as fishing, swimming, and canoeing are conducted in some areas of these wetlands (54).

### Sampling sites.

Sampling sites were located in landscape units IIb and IV of the noninsular portions of the Lower Delta (54) (Fig. 1). These units are characterized by gentle undulations due to the presence of sandy ridges separated by depressions. Sampling sites were located either in the lower portion of the topographical gradient (freshwater marshlands) or in ditches within polderized livestock fields. Sampling sites were characterized by sediments rich in organic matter over a dense horizon of clay and fine silt content. Ditches were mainly characterized by free-floating macrophytes, mostly represented by Eichhornia crassipes, *Salvinia* spp., Azolla filiculoides, Pistia stratiotes, Limnobium laevigatum, and *Lemna* spp. In contrast, freshwater marshlands were mainly characterized by their most conspicuous species, Schoenoplectus californicus, as well as by accompanying emergent macrophytes such as Ludwigia peploides, Enhydra anagallis, *Sagittaria* sp., Myriophyllum aquaticum, and *Pontederia* spp. and the free-floating species mentioned above. In some cases, submerged vegetation, including algae and vascular plants such as Ceratophyllum demersum, were present as well. Samplings were conducted in November 2018 (spring), April 2019 (autumn), and November 2019 (spring) across nine freshwater marshlands (S1, S2, S3, S4, S5a, S5b, S6, S7, S8, and S9) and six ditches (S10, S11, S12, S13, S14, and S15) across livestock fields subject to permanent livestock raising and differing livestock management practices (Fig. 1). Due to the larger extension of freshwater marshland S5, and in order to consider its internal heterogeneity, two subsamples were collected (S5a and S5b).

### Hydrometeorological conditions.

In order to assess and describe the hydrometeorological conditions for each sampling period, we selected four variables that serve as indicators of the hydrologic regimen and water availability in the study area through the years 2018 and 2019: multivariate ENSO index, monthly mean precipitation, land surface temperature, and hydrometric levels of the Paranacito River. The MEI v2 data (56), provided by the Physical Sciences Laboratory (https://psl.noaa.gov/enso/mei/), identify the occurrence of El Niño (wet)/La Niña (dry) cycles and intensities. Due to the lack of precipitation-measuring instruments on the field, monthly precipitation data were obtained from the Integrated Multi-satellite Retrievals for GPM (IMERG) (57). Monthly values of land surface temperature were estimated from the MOD11C3 product (58). Water level time series data for the Paranacito River between 2018 and 2019 were provided by Prefectura Naval Argentina (https://contenidosweb.prefecturanaval.gob.ar/alturas/). In order to properly assess the hydrometeorological conditions for each sampling period, data covering the 3 months prior to each sampling date were considered.

### Sample collection.

Five subsurface water samples were collected at each sampling site and period, in sterile 50-mL plastic Falcon tubes. During November 2019, a period of extremely low water level occurred, which might increase the risk of human and animal exposure to sediment microorganisms as a consequence of the reduced water column. Therefore, in addition to water samples, approximately 5 g of sediment was collected during this period by using sterile 50-mL plastic Falcon tubes.

Sampling design was conditioned by the accessibility and flooding conditions at each sampling site. In case of floods due to heavy rain or river overflow events that impeded access to sampling sites, no samples were collected.

### Culture of leptospires.

Culture of *Leptospira* was performed immediately upon sample collection. For each water sample, 1 mL was filtered through a sterile membrane (0.22-μm pore size). The filtered water was inoculated into liquid and semisolid Ellinghausen-McCullough-Johnson-Harris (EMJH) medium (Difco Laboratories, Detroit, MI, USA) with the addition of 5-fluorouracil (FLU) (300 μg/mL) as a selective antimicrobial agent. Sediment culture was performed by adding 20 mL sterile distilled water to each plastic tube, shaking vigorously, and allowing sediment to settle for 15 min. Approximately 1 mL of the supernatant was taken and filtered with a sterile membrane with 0.22-μm pores. Two drops of the filtrate was inoculated into the semisolid EMJH medium. Cultures were incubated at 28°C, and leptospiral growth was monitored weekly for up to 4 months using dark-field microscopy. After 4 months without the presence of *Leptospira*, the culture was considered negative and discarded. In the case of sample contamination, 1 mL of the old culture was extracted and filtered into new EMJH medium with the addition of FLU (300 μg/mL), using a 0.22-μm-pore-size membrane syringe filter.

### DNA extraction.

All samples were maintained and transported to the laboratory on ice for protection against sudden temperature changes and processed within 12 h after collection. Both water and sediment samples were centrifuged at 3,000 rpm (relative centrifugal force [RCF], 100) for 5 min. The supernatant was recovered and centrifuged at 8,000 rpm (RCF, 12,000) for 30 min at 4°C. Pellets of each quintuplicate sample were pooled, and then DNA was extracted using the QIAamp DNA minikit (Qiagen, Valencia, CA) following the manufacturer’s instructions.

### 16S rRNA.

Determination of species was performed using 16S rRNA as the amplification target (59). For each reaction, 1 U of GoTaq DNA polymerase (Promega, Madison, WI, USA), 200 μM deoxynucleoside triphosphates (dNTPs), and 1 μM primers were added to a total volume of 50 μL. Amplification was carried out using a Veriti thermal cycler (Applied Biosystems, Foster City, CA, USA), and the PCR products were analyzed on 2% agarose gels.

### Sequencing and sequence analysis.

PCR amplification products of 16S rRNA were purified using a GeneJET PCR purification kit (Thermo Scientific, Waltham, MA, USA) prior to DNA sequencing. PCR products were then sequenced by Macrogen Inc. (Seoul, South Korea). The sequences were edited using Chromas Lite 2.1.1 (Technelysium Pty., Ltd., Australia). The contigs were assembled using Staden package software (MRC-LMB, United Kingdom), and the alignment was performed using MEGA 5 (60). To obtain the *Leptospira* species, the assembled sequences of 16S rRNA were analyzed using the Ribosomal Database Project (RDP) (http://rdp.cme.msu.edu/).

### Physicochemical analysis.

Water temperature, dissolved oxygen, conductivity, and pH were measured *in situ* with Hanna portable checkers. Subsurface water samples were collected in duplicates at each sampling site. Samples were filtered through Whatman GF/C glass fiber filters (pore size, 1.2 μm) within 24 h after sampling. Filters were stored at −20°C up to 3 weeks for spectrophotometric analysis of chlorophyll *a*, which was extracted with 90% acetone according to Lorenzen’s method (61).

Filtered samples were passed through Millipore filters (pore size, 0.45 μm) and kept frozen until spectrophotometric determination of dissolved nutrients following (61). Nitrite (NO_2_^−^) was determined by diazotizing with sulfanilamide and coupling with *N*-(1-naphthyl)-ethylenediamine dihydrochloride, nitrate plus nitrite (NO_3_^−^ + NO_2_^−^) was assessed by reduction of nitrate with hydrazine sulfate and subsequent determination of nitrite, ammonium (NH_4_^+^) was assessed by the indophenol blue method, and soluble reactive phosphorus (SRP) was assessed by the ascorbic acid method. The concentration of nitrate (NO_3_^−^) was calculated from the difference between NO_3_^−^ + NO_2_^−^ and NO_2_^−^. Total phosphorus (TP) and total nitrogen (TN) were estimated from unfiltered water samples that were kept frozen until analysis. TP was estimated by digestion with nitric and sulfuric acids followed by SRP determination, and TN was estimated by digestion with potassium persulfate in alkaline medium followed by NO_3_^−^ + NO_2_^−^ determination (61). An aliquot of each water sample filtered through 0.45-μm filters was kept in darkness and refrigerated (4°C) until optical analysis of CDOM. Absorbance at 440 and 700 nm was measured using 1-cm quartz cuvettes and filtered Milli-Q water as a baseline. The absorbance at 700 nm was subtracted from the absorbance at 440 nm to correct offsets (62). The absorption coefficient (m^−1^) at 440 nm (*A*_440_) was calculated according to (63) from the corrected absorbance at this wavelength and used as a measure of CDOM concentration. All the spectrophotometric determinations were carried out using a Shimadzu UV-1800 UV/visible-light spectrophotometer.

### Data analysis.

Patterns of spatiotemporal variability of physical and chemical variables were assessed via PCA, using the software CANOCO version 5 (Microcomputer Power, Ithaca, NY, USA). Variables were log transformed (except pH due to its logarithmic nature), centered, and standardized prior to the analysis. Comparisons among the three sampling periods for each physical and chemical variable were performed using repeated-measures ANOVA. Data were Box-Cox transformed when needed to fit homogeneity of variance assumption.

## References

[1] Ko AI, Goarant C, Picardeau M. 2009. Leptospira: the dawn of the molecular genetics era for an emerging zoonotic pathogen. Nat Rev Microbiol 7:736–747. doi:10.1038/nrmicro2208. 19756012 PMC3384523

[2] Levett PN. 2001. Leptospirosis. Clin Microbiol Rev 14:296–326. doi:10.1128/CMR.14.2.296-326.2001. 11292640 PMC88975

[3] Picardeau M. 2020. Leptospira and leptospirosis. Methods Mol Biol 2134:271–275. doi:10.1007/978-1-0716-0459-5_24. 32632877

[4] Vincent AT, Schiettekatte O, Goarant C, Neela VK, Bernet E, Thibeaux R, Ismail N, Mohd Khalid MKN, Amran F, Masuzawa T, Nakao R, Amara Korba A, Bourhy P, Veyrier FJ, Picardeau M. 2019. Revisiting the taxonomy and evolution of pathogenicity of the genus Leptospira through the prism of genomics. PLoS Negl Trop Dis 13:e0007270. doi:10.1371/journal.pntd.0007270. 31120895 PMC6532842

[5] Adler B, de la Peña Moctezuma A. 2010. Leptospira and leptospirosis. Vet Microbiol 140:287–296. doi:10.1016/j.vetmic.2009.03.012. 19345023

[6] Bierque E, Thibeaux R, Girault D, Soupé-Gilbert ME, Goarant C. 2020. A systematic review of Leptospira in water and soil environments. PLoS One 15:e0227055. doi:10.1371/journal.pone.0227055. 31986154 PMC6984726

[7] Haake DA, Levett PN. 2015. Leptospirosis in humans. Curr Top Microbiol Immunol 387:65–97. doi:10.1007/978-3-662-45059-8_5. 25388133 PMC4442676

[8] Monahan AM, Callanan JJ, Nally JE. 2009. Review paper: Host-pathogen interactions in the kidney during chronic leptospirosis. Vet Pathol 46:792–799. doi:10.1354/vp.08-VP-0265-N-REV. 19429975

[9] Thibeaux R, Soupé-Gilbert M-E, Kainiu M, Girault D, Bierque E, Fernandes J, Bähre H, Douyère A, Eskenazi N, Vinh J, Picardeau M, Goarant C. 2020. The zoonotic pathogen Leptospira interrogans mitigates environmental stress through cyclic-di-GMP-controlled biofilm production. NPJ Biofilms Microbiomes 6:24. doi:10.1038/s41522-020-0134-1. 32532998 PMC7293261

[10] Bulach DM, Zuerner RL, Wilson P, Seemann T, McGrath A, Cullen PA, Davis J, Johnson M, Kuczek E, Alt DP, Peterson-Burch B, Coppel RL, Rood JI, Davies JK, Adler B. 2006. Genome reduction in Leptospira borgpetersenii reflects limited transmission potential. Proc Natl Acad Sci USA 103:14560–14565. doi:10.1073/pnas.0603979103. 16973745 PMC1599999

[11] Goarant C, Trueba G, Bierque E, Thibeaux R, Davis B, de la Pena-Moctezuma A. 2019. Leptospira and leptospirosis. *In*: Rose JB, Jiménez Cisneros B, UNESCO International Hydrological Programme (ed), Water and sanitation for the 21st century: health and microbiological aspects of excreta and wastewater management (Global Water Pathogen Project). Michigan State University, East Lansing, MI.

[12] Zarantonelli L, Suanes A, Meny P, Buroni F, Nieves C, Salaberry X, Briano C, Ashfield N, Da Silva Silveira C, Dutra F, Easton C, Fraga M, Giannitti F, Hamond C, Macías-Rioseco M, Menéndez C, Mortola A, Picardeau M, Quintero J, Ríos C, Rodríguez V, Romero A, Varela G, Rivero R, Schelotto F, Riet-Correa F, Buschiazzo A, Grupo de Trabajo Interinstitucional de Leptospirosis Consortium. 2018. Isolation of pathogenic Leptospira strains from naturally infected cattle in Uruguay reveals high serovar diversity, and uncovers a relevant risk for human leptospirosis. PLoS Negl Trop Dis 12:e0006694. doi:10.1371/journal.pntd.0006694. 30212451 PMC6136691

[13] Azali MA, Yean Yean C, Harun A, Aminuddin Baki NN, Ismail N. 2016. Molecular characterization of Leptospira spp. in environmental samples from North-Eastern Malaysia revealed a pathogenic strain, Leptospira alstonii. J Trop Med 2016:2060241. doi:10.1155/2016/2060241. 27127522 PMC4834157

[14] Dzulaikha K, Nurul Yuziana MY, Maizatulriah JJ, Marfiah AW. 2018. Association of rainfall and the occurrence of pathogenic Leptospira spp. in recreational stream water, Hulu Langat, Selangor, p 119–124. *In* Ibrahim F, Usman J, Ahmad MY, Hamzah N, Teh SJ (ed), 2nd International Conference for Innovation in Biomedical Engineering and Life Sciences (IFMBE Proceedings). Springer, Singapore.

[15] Thibeaux R, Geroult S, Benezech C, Chabaud S, Soupé-Gilbert M-E, Girault D, Bierque E, Goarant C. 2017. Seeking the environmental source of Leptospirosis reveals durable bacterial viability in river soils. PLoS Negl Trop Dis 11:e0005414. doi:10.1371/journal.pntd.0005414. 28241042 PMC5344526

[16] Schneider AG, Casanovas-Massana A, Hacker KP, Wunder EA, Begon M, Reis MG, Childs JE, Costa F, Lindow JC, Ko AI. 2018. Quantification of pathogenic Leptospira in the soils of a Brazilian urban slum. PLoS Negl Trop Dis 12:e0006415. doi:10.1371/journal.pntd.0006415. 29624576 PMC5906024

[17] Jagai JS, Griffiths JK, Kirshen PK, Webb P, Naumova EN. 2012. Seasonal patterns of gastrointestinal illness and streamflow along the Ohio River. Int J Environ Res Public Health 9:1771–1790. doi:10.3390/ijerph9051771. 22754472 PMC3386587

[18] Pettit NE, Jardine TD, Hamilton SK, Sinnamon V, Valdez D, Davies PM, Douglas MM, Bunn SE. 2012. Seasonal changes in water quality and macrophytes and the impact of cattle on tropical floodplain waterholes. Mar Freshw Res 63:788. doi:10.1071/MF12114.

[19] Mayora G, Piedrabuena A, Ferrato JJ, Gutierrez MF, Mesa L. 2021. Water quality dynamics of floodplain lakes in relation to river flooding and cattle grazing. Mar Freshw Res 72:1496–1505. doi:10.1071/MF20297.

[20] Trueba G, Zapata S, Madrid K, Cullen P, Haake D. 2004. Cell aggregation: a mechanism of pathogenic Leptospira to survive in fresh water. Int Microbiol 7:35–40. 15179605

[21] de Oliveira D, Airam Querino V, Sara Lee Y, Cunha M, Nery N, Jr, Wessels Perelo L, Rossi Alva JC, Ko AI, Reis MG, Casanovas-Massana A, Costa F. 2020. Relationship between physicochemical characteristics and pathogenic Leptospira in urban slum waters. Trop Med 5:146. doi:10.3390/tropicalmed5030146. PMC7558472 32947807

[22] Narkkul U, Thaipadungpanit J, Srilohasin P, Singkhaimuk P, Thongdee M, Chaiwattanarungruengpaisan S, Krairojananan P, Pan-Ngum W. 2020. Optimization of culture protocols to isolate Leptospira spp. from environmental water, field investigation, and identification of factors associated with the presence of Leptospira spp. in the environment. Trop Med 5:94. doi:10.3390/tropicalmed5020094. PMC7345561 32517121

[23] Barragan VA, Mejia ME, Trávez A, Zapata S, Hartskeerl RA, Haake DA, Trueba GA. 2011. Interactions of Leptospira with environmental bacteria from surface water. Curr Microbiol 62:1802–1806. doi:10.1007/s00284-011-9931-3. 21479795

[24] Benacer D, Woh PY, Mohd Zain SN, Amran F, Thong KL. 2013. Pathogenic and saprophytic Leptospira species in water and soils from selected urban sites in peninsular Malaysia. Microbes Environ 28:135–140. doi:10.1264/jsme2.me12154. 23363618 PMC4070680

[25] Saito M, Villanueva SYAM, Chakraborty A, Miyahara S, Segawa T, Asoh T, Ozuru R, Gloriani NG, Yanagihara Y, Yoshida S-I. 2013. Comparative analysis of Leptospira strains isolated from environmental soil and water in the Philippines and Japan. Appl Environ Microbiol 79:601–609. doi:10.1128/AEM.02728-12. 23144130 PMC3553789

[26] Rawlins J, Portanova A, Zuckerman I, Loftis A, Ceccato P, Willingham AL, Verma A. 2014. Molecular detection of leptospiral DNA in environmental water on St. Kitts. Int J Environ Res Public Health 11:7953–7960. doi:10.3390/ijerph110807953. 25105546 PMC4143842

[27] Quintana RD, Bó RF. 2010. Caracterización general de la región del Delta del Paraná, p. 5–13. *In* Blanco DE, Méndez FM. Endicamientos y terraplenes en el Delta del Paraná. Situación, efectos ambientales y marco jurídico. Fundación Humedales/Wetlands International. Buenos Aires, Argentina.

[28] Malvárez AI. 1999. El Delta del Río Paraná como mosaico de humedales. Tópicos Humed Subtrop Templados Sudam 1:35–54.

[29] Quintana RD, Bó RF, Astrada E, Reeves C. 2014. Lineamientos para una ganadería ambientalmente sustentable en el Delta del Paraná. Fundación Humedales–Wetlands International, Buenos Aires, Argentina.

[30] Sica YV, Quintana RD, Radeloff VC, Gavier-Pizarro GI. 2016. Wetland loss due to land use change in the Lower Paraná River Delta, Argentina. Sci Total Environ 568:967–978. doi:10.1016/j.scitotenv.2016.04.200. 27369090

[31] Lall C, Kumar KV, Raj RV, Vedhagiri K, Vijayachari P. 2016. Prevalence and diversity of leptospires in different ecological niches of urban and rural areas of South Andaman Island. Microbes Environ 31:79–82. doi:10.1264/jsme2.ME15149. 26936796 PMC4791121

[32] Yusof NY, Muhammad Yusoff F, Muhammad Harish S, Ahmad MN, Khalid MF, Mohd Nor F, Ismail N, Aziah I. 2019. Complete genome sequence of Leptospira kmetyi LS 001/16, isolated from a soil sample associated with a leptospirosis patient in Kelantan, Malaysia. Microbiol Resour Announc 8:e00015-19. doi:10.1128/MRA.00015-19. 31296668 PMC6624751

[33] Sato Y, Hermawan I, Kakita T, Okano S, Imai H, Nagai H, Kimura R, Yamashiro T, Kajita T, Toma C. 2022. Analysis of human clinical and environmental Leptospira to elucidate the eco-epidemiology of leptospirosis in Yaeyama, subtropical Japan. PLoS Negl Trop Dis 16:e0010234. doi:10.1371/journal.pntd.0010234. 35358181 PMC8970387

[34] Bourhy P, Collet L, Brisse S, Picardeau M. 2014. Leptospira mayottensis sp. nov., a pathogenic species of the genus Leptospira isolated from humans. Int J Syst Evol Microbiol 64:4061–4067. doi:10.1099/ijs.0.066597-0. 25249563 PMC4811635

[35] Wilkinson DA, Edwards M, Benschop J, Nisa S. 2021. Identification of pathogenic Leptospira species and serovars in New Zealand using metabarcoding. PLoS One 16:e0257971. doi:10.1371/journal.pone.0257971. 34587213 PMC8480790

[36] Weinberger D, Baroux N, Grangeon JP, Ko AI, Goarant C. 2014. El Niño southern oscillation and leptospirosis outbreaks in New Caledonia. PLoS Negl Trop Dis 8:e2798. doi:10.1371/journal.pntd.0002798. 24743322 PMC3990495

[37] Aquino D, Gavier-Pizarro GI, Quintana RD. 2021. Disentangling the effects of hydro-climatic factors and land use intensification on wetland vegetation dynamics in the Lower Delta of the Paraná River. Remote Sens Applic Soc Environ 21:100466. doi:10.1016/j.rsase.2021.100466.

[38] Thaipadungpanit J, Wuthiekanun V, Chantratita N, Yimsamran S, Amornchai P, Boonsilp S, Maneeboonyang W, Tharnpoophasiam P, Saiprom N, Mahakunkijcharoen Y, Day NPJ, Singhasivanon P, Peacock SJ, Limmathurotsakul D. 2013. Leptospira species in floodwater during the 2011 floods in the Bangkok metropolitan region, Thailand. Am J Trop Med Hyg 89:794–796. doi:10.4269/ajtmh.13-0124. 24002484 PMC3795115

[39] Ghizzo Filho J, Nazário NO, Freitas PF, Pinto de GA, Schlindwein AD. 2018. Temporal analysis of the relationship between leptospirosis, rainfall levels and seasonality, Santa Catarina, Brazil, 2005–2015. Rev Inst Med Trop São Paulo 60:e39. doi:10.1590/S1678-9946201860039. 30066807 PMC6069269

[40] Dutra F, Valadão RC, Confalonieri UE, Muller GV, de Quadro MFLA. 2015. Influência da variabilidade da precipitação no padrão de distribuição dos casos de leptospirose em Minas Gerais no período de 1998 a 2012. https://ri.conicet.gov.ar/handle/11336/69327.

[41] Chadsuthi S, Chalvet-Monfray K, Wiratsudakul A, Modchang C. 2021. The effects of flooding and weather conditions on leptospirosis transmission in Thailand. Sci Rep 11:1486. doi:10.1038/s41598-020-79546-x. 33452273 PMC7810882

[42] Mayora G, Schneider B, Rossi A. 2018. Turbidity and dissolved organic matter as significant predictors of spatio-temporal dynamics of phosphorus in a large river-floodplain system: phosphorus dynamics in a river-floodplain system. River Res Applic 34:629–639. doi:10.1002/rra.3288.

[43] Villar CA, Bonetto C. 2000. Chemistry and nutrient concentrations of the Lower Parana River and its floodplain marshes during extreme flooding. Fundam Appl Limnol 148:461–479. doi:10.1127/archiv-hydrobiol/148/2000/461.

[44] Vinod Kumar K, Lall C, Raj RV, Vedhagiri K, Sunish IP, Vijayachari P. 2016. In vitro antimicrobial susceptibility of pathogenic Leptospira biofilm. Microb Drug Resist 22:511–514. doi:10.1089/mdr.2015.0284. 26978023

[45] Simm R, Morr M, Kader A, Nimtz M, Römling U. 2004. GGDEF and EAL domains inversely regulate cyclic di-GMP levels and transition from sessility to motility. Mol Microbiol 53:1123–1134. doi:10.1111/j.1365-2958.2004.04206.x. 15306016

[46] Ristow P, Bourhy P, Kerneis S, Schmitt C, Prevost M-C, Lilenbaum W, Picardeau M. 2008. Biofilm formation by saprophytic and pathogenic leptospires. Microbiology 154:1309–1317. doi:10.1099/mic.0.2007/014746-0. 18451039

[47] Scialfa E, Grune S, Brihuega Aguirre P, Rivero M. 2018. Isolation of saprophytic Leptospira spp. from a selected environmental water source of Argentina. Rev Argent Microbiol 50:323–326. 29198840 10.1016/j.ram.2017.08.003

[48] Masuzawa T, Sakakibara K, Saito M, Hidaka Y, Villanueva SYAM, Yanagihara Y, Yoshida S-I. 2018. Characterization of Leptospira species isolated from soil collected in Japan. Microbiol Immunol 62:55–59. doi:10.1111/1348-0421.12551. 29105847

[49] Chaiwattanarungruengpaisan S, Suwanpakdee S, Sangkachai N, Chamsai T, Taruyanon K, Thongdee M. 2018. Potentially pathogenic Leptospira species isolated from a waterfall in Thailand. Jpn J Infect Dis 71:65–67. doi:10.7883/yoken.JJID.2017.363. 29093324

[50] Chakraborty A, Miyahara S, Villanueva SYAM, Saito M, Gloriani NG, Yoshida S. 2011. A novel combination of selective agents for isolation of Leptospira species. Microbiol Immunol 55:494–501. doi:10.1111/j.1348-0421.2011.00347.x. 21545510

[51] Lilenbaum W, Martins G. 2014. Leptospirosis in cattle: a challenging scenario for the understanding of the epidemiology. Transbound Emerg Dis 61:63–68. doi:10.1111/tbed.12233. 25135465

[52] Zaki AM, Hod R, Shamsusah NA, Isa ZM, Bejo SK, Agustar HK. 2020. Detection of Leptospira kmetyi at recreational areas in Peninsular Malaysia. Environ Monit Assess 192:703. doi:10.1007/s10661-020-08639-x. 33057929

[53] Quintana RD, Bó RF. 2011. ¿Por qué el Delta del Paraná es una región única?, p. 42–53. *In* Quintana R, Villar V, Astrada E, Sacconey P, Malsof S (ed), El patrimonio natural y cultural del Bajo Delta Insular. Bases para su conservación y uso sustentable. Aprendelta/Convensión Internacional sobre los Humedales (Ramsar, Irán, 1971). Buenos Aires, Argentina.

[54] Kandus P, Quintana RD, Bó RF. 2006. Land scape patterns and biodiversity of the lower delta of the Paraná River. 2006; mapa de ambientes, p 48. Pablo Casamajor Ediciones, Buenos Aires, Argentina.

[55] Minotti PG. 2019. Actualización y profundización del mapa de endicamientos y terraplenes de la región del Delta del Paraná. Informe actualización 2018. Fundación para la Conservación y el Uso Sustentable de los Humedales/Wetlands International. Buenos Aires, Argentina.

[56] Zhang H, Zhang C, Zhu Y, Mehmood K, Liu J, McDonough SP, Tang Z, Chang Y-F. 2020. Leptospirosis trends in China, 2007–2018: a retrospective observational study. Transbound Emerg Dis 67:1119–1128. doi:10.1111/tbed.13437. 31765064

[57] Huffman G, Bolvin D, Nelkin E, Tan J. 2019. Integrated Multi-satellitE Retrievals for GPM (IMERG) technical documentation. https://gpm.nasa.gov/sites/default/files/document_files/IMERG_doc.pdf.

[58] Wan Z, Hook S, Hulley G. 2015. MOD11B3 MODIS/Terra Land Surface Temperature/Emissivity Monthly L3 Global 6 km SIN Grid V006 Income. https://earthexplorer.usgs.gov.

[59] Mérien F, Amouriaux P, Perolat P, Baranton G, Saint Girons I. 1992. Polymerase chain reaction for detection of Leptospira spp. in clinical samples. J Clin Microbiol 30:2219–2224. doi:10.1128/jcm.30.9.2219-2224.1992. 1400983 PMC265482

[60] Tamura K, Peterson D, Peterson N, Stecher G, Nei M, Kumar S. 2011. MEGA5: molecular evolutionary genetics analysis using maximum likelihood, evolutionary distance, and maximum parsimony methods. Mol Biol Evol 28:2731–2739. doi:10.1093/molbev/msr121. 21546353 PMC3203626

[61] APHA. 2017. Standard methods for the examination of water and wastewater, 23rd ed, p 700. APHA, Washington, DC.

[62] Green SA, Blough NV. 1994. Optical absorption and fluorescence properties of chromophoric dissolved organic matter in natural waters. Limnol Oceanogr 39:1903–1916. doi:10.4319/lo.1994.39.8.1903.

[63] Kirk JTO. 1994. Light and photosynthesis in aquatic ecosystems, p 530. Cambridge University Press, Cambridge, United Kingdom.

